# Optimization of Early Antimicrobial Strategies for Lung Transplant Recipients Based on Metagenomic Next-Generation Sequencing

**DOI:** 10.3389/fmicb.2022.839698

**Published:** 2022-03-23

**Authors:** Xiao-qin Zhang, Yu Lei, Xiao-li Tan, Lu Guo, Xiao-bo Huang, Fu-xun Yang, Hua Yu, Xiao-shu Liu, Yi-ping Wang, Sen Lu, Ling-ai Pan

**Affiliations:** ^1^Department of Critical Care Medicine, Sichuan Academy of Medical Sciences and Sichuan Provincial People’s Hospital, Chengdu, China; ^2^Genoxor Medical Science and Technology Inc., Taizhou, China; ^3^Department of Pulmonary and Critical Care Medicine, Sichuan Academy of Medical Sciences and Sichuan Provincial People’s Hospital, Chengdu, China; ^4^Department of Microbiology Laboratory, Sichuan Academy of Medical Sciences and Sichuan Provincial People’s Hospital, Chengdu, China

**Keywords:** metagenomic next-generation sequencing, lung tissue, lung transplantation, bronchoalveolar lavage fluid, pathogen detection

## Abstract

The management of perioperative antibiotic options after lung transplantation varies widely around the world, but there is a common trend to limit antibiotic use duration. Metagenomic next-generation sequencing (mNGS) has become a hot spot in clinical pathogen detection due to its precise, rapid, and wide detection spectrum of pathogens. Thus, we defined a new antibiotic regimen adjustment strategy in the very early stage (within 7 days) after lung transplantation mainly depending on mNGS reports combined with clinical conditions to reduce the use of antibiotics. To verify the clinical effect of the strategy, we carried out this research. Thirty patients who underwent lung transplantation were finally included, whose information including etiology, antibiotic adjustment, and the effect of our strategy was recorded. Lung transplant recipients in this study were prescribed with initial antibiotic regimen immediately after surgery; their antibiotic regimens were adjusted according to the strategy. According to our study, the entire effectiveness of the strategy was 90.0% (27/30). Besides, a total of 86 samples containing donor lung tissue, recipient lung tissue, and bronchoalveolar lavage fluid (BALF) were obtained in this study; they were all sent to mNGS test, while BALF was also sent to pathogen culture. Their results showed that the positive rate of BALF samples was higher (86.67%) than that of donor’s lung tissue (20.0%) or recipient’s lung tissue (13.33%) by mNGS test, indicating BALF samples are more valuable than other clinical samples from early postoperative period to guide the early adjustment of antibiotics after lung transplantation. It is effective for mNGS combined with traditional methods and clinical situations to optimize antibiotic regimens in lung transplantation recipients within 7 days after surgery.

## Introduction

Infectious complications remain a significant cause of morbidity and mortality in lung transplant recipients (Burguete et al., 2013; Raskin et al., 2020), who are at increased risk of infection for multiple reasons, including continuous exposure of the allograft to environmental microorganisms and denervation of the lung resulting in impaired cough reflex, dysfunctional mucociliary clearance, impaired lymphatic drainage, and immunosuppression (Costa et al., 2017).

At present, broad-spectrum antibiotics are used to cover the infection after lung transplantation worldwide, and hospital-acquired bacteria are mostly targeted by perioperative antibiotic therapy even if no pathogen colonization occurs (Coiffard et al., 2020). However, this may cause problems of antibiotic abuse and resistance, and also potential liver and kidney injury. Therefore, precise anti-infective treatment during the perioperative period is particularly important.

A study conducted by Coiffard et al. (2020) analyzed worldwide clinical practices in perioperative antibiotic therapy for lung transplantation; they collected data from 99 hospitals in 24 countries, and the result showed that the duration of prophylaxis in this context was heterogeneous but mostly 7 days (33.3%) or shorter (26.3%), or until cultures of the donor and the recipients were reported as negative (12.1%). The antibiotic treatment was almost systematically adapted to the results of the donor samples (97.1%). After 4 days of empirical treatment, if the results of the bacteriological screening were negative, and there was no sign of infection, antibiotics were stopped in 52.5% of the centers. This suggests that the antibiotic regimen in the very early stage (within 7 days) after lung transplantation is very important.

Precise pathogen detection is a prerequisite for precise anti-infection treatment (Zhou et al., 2019). Traditional pathogen testing methods cannot meet clinical needs due to the disadvantages of a long detection cycle and low sensitivity. Metagenomic next-generation sequencing (mNGS) could yield higher sensitivity (Chen et al., 2020; Duan et al., 2021) for pathogen identification and is less affected by prior antibiotic exposure (Miao et al., 2018; Huang et al., 2020), thereby emerging as a promising technology for detecting infectious diseases (Miao et al., 2018; Li et al., 2020; Duan et al., 2021). Two previous studies (Liu et al., 2020; Lian et al., 2021) found that mNGS is effective in detecting pathogens after lung transplantation. mNGS combined with traditional methods and pathogen detection of different samples including donor lung tissue, recipient lung tissue, and BALF immediately after surgery can theoretically be more comprehensive obtaining the background information of the pathogen and making anti-infective treatment more targeted. At the same time, because mNGS is short time-consuming, the results can be obtained quickly, even if the infection occurs in the ultra-early postoperative period. Adjusting the antibiotics according to the pathogen detection results in advance will be more targeted; this approach is similar to preemptive therapy in anti-fungal therapy.

Therefore, we developed a new strategy for super early-stage (within 7 days) antibiotic optimization after lung transplantation: donor lung tissue, recipient lung tissue, and BALF within 2 h after surgery were all sent to mNGS (BALF samples were also sent to pathogen culture). We gave the patients a basic antibiotic regimen (relatively narrow spectrum) soon after the operation and optimized the regimen according to their clinical indications, and pathogen detection results after pathogen detection reports were obtained.

The purpose of this study is to validate the clinical practical effect of the above antibiotic adjustment strategy; evaluate mNGS combined with traditional methods to detect donor lung tissue, receptor lung tissue, and BALF; and guide the adjustment of early antibacterial strategies, determine whether they can affect the early prognosis of lung transplantation recipients, and provide a clinical reference for anti-infection work in the super early stage post lung transplantation.

## Materials and Methods

### Criteria for Donor Lungs Inclusion and Exclusion

Donor lungs inclusion criteria: (a) Age < 60 years old, smoking history < 20 packs/year. (b) No chest injury. (c) Continuous mechanical ventilation < 1 week. (d) PaO_2_ > 300 mmHg (FiO_2_ = 100%, PEEP = 5 cmH_2_O). (e) X-ray or CT shows that the lung field is relatively clear. (f) There is no abscess secretion in the lung bronchus at all levels through bronchoscopy (Liu et al., 2020).

Donor lungs exclusion criteria: (a) Age > 60 years old, smoking history > 20 packs/year. (b) Chest trauma and lung contusion. (c) Continuous mechanical ventilation > 1 week. (d) PaO_2_ < 300 mmHg (FiO_2_ = 100%, PEEP = 5 cmH_2_O). (e) X-ray or CT shows that the lung field is infected. (f) There are purulent secretions at bronchoscopy in the donor lower airways. (g) The percentage of white blood cells, neutrophils, C-reactive protein, and procalcitonin increases gradually compared with the situation at the onset of the disease. (h) The donor’s body temperature is higher than normal. (i) Blood culture is positive.

### Data Acquisition and Samples Collection

Patients undergoing lung transplantation at Sichuan Provincial People’s Hospital from October 2018 to July 2021 were included in this study, and baseline data (at the time of admission) and data during the hospitalization period were collected. Baseline data mainly include gender, age, date of admission, underlying disease information, basic lung pathogen colonization, and infection information.

After enrollment, we record information about lung transplantation including donor information, single or bilateral lung transplantation, and sampling protocols as follows: BALF samples from recipients were obtained within 2 h after surgery, which must be submitted to traditional pathogen culture and mNGS pathogen detection at the same time. The donor lung tissue samples from the basal segment of the lower lobe with a size of 0.5 cm × 0.5 cm were sent to mNGS test. Lung tissue samples of the recipients from the basal segment of the lower lobe with a size of 0.5 cm × 0.5 cm were also sent to mNGS test.

Prognostic information on antimicrobial use of the enrolled patients and prognostic information on mechanical ventilation, ICU hospitalization, new-onset infection, and 30-day mortality were recorded after surgery.

### mNGS Sequencing and Data Analysis

The samples were stored at 4°C and sent to Genoxor Medical Laboratory for mNGS detection within 24 h. The 1.5-ml microcentrifuge tube with the 0.6-ml (/g) sample, enzyme, and 1.0 g of 0.5-mm glass beads was attached to a horizontal platform on a vortex mixer and agitated vigorously at 2,800–3,200 rpm for 30 min. Then the 0.3-mL sample was separated into new 1.5-mL microcentrifuge tubes, and DNA of BALF samples was extracted using the TIANamp Micro DNA Kit (DP316, Tiangen Biotech) according to the manufacturer’s instructions. DNA of lung tissue samples was extracted using the TIANamp Genomic DNA Kit (DP304, Tiangen Biotech) according to the manufacturer’s instructions. Then, DNA libraries were constructed through DNA fragmentation, end-repair, adapter ligation, and PCR amplification. Agilent 2100 was used for quality control of the DNA libraries. Library concentration was measured by Qubit 2.0, and sequencing data was pre-quantified by q-PCR. Quality-qualified libraries were sequenced on the NextSeq™ 550DX platform in SE-75 sequencing type according to the manufacturer’s instructions.

Raw data were split using bcl2fastq2 software, and the connector sequences and low-quality base sequences were removed using Trimmomatic software to obtain high-quality effective data. Sequences from the human genome were removed using the bowtie2 calibration software. Eventually, sequences that could not be mapped to the human genome were retained and the rest of the sequences were aligned to the microbial genome database, which was constructed using the sequences of bacteria, fungi, archaea, and viruses screened in the NCBI database, covering 16,834 microbes (7,982 bacteria, 7,811 viruses, and 124 parasites).

To enable a comparison between species within the same sample, the number of reads was homogenized. The numbers of reads were normalized with the genome length to calculate their RPK (reads per kilobase), and the species’ relative abundance was further calculated based on the RPK.

### Antibiotic Regimen Adjustment Strategy

An initial antimicrobial protocol was formulated based on the donor, recipient, and perioperative conditions. The principle of the initial protocol is: cover all the possible pathogens with minimal antimicrobial drugs.

After the traditional pathogen detection and mNGS reports were acquired, the adjustment of the antibiotic regimen was made according to the following conditions:

(I) Maintain the basic antibiotic regimen. (II) Change the basic antibiotic regimen based on the clinical characteristics and pathogen detection results of patients: (II-1) Antimicrobial de-escalation (ADE) treatment or simplified antibiotic regimen because of no signs of infection; (II-2) There were no new-onset infections but we add some other antibiotics in advance or replace the original antibiotics according to the clinical characteristics and pathogen detection results; (II-3) There were new-onset infections and we change the antibiotics.

### Effectiveness Evaluation of Antibiotic Adjustment Strategy

The effectiveness judgment of the antibiotic strategy was made according to the new-onset infections and other clinical indicators, such as MV time and ICU hospitalization. This effectiveness evaluation requires at least two transplant management experts. The effect of the strategy was determined as “Positive,” “Negative,” and “None,” as indicated respectively:

“Positive”: The patients have no new-onset infections within 1 week after surgery, or effectively controlled infection after adjusting antibiotic regimen by referring to the mNGS positive results.

“Negative”: There were new-onset infections and aggravation, even if the antimicrobial regimen is optimized based on mNGS reports.

“None”: The role of mNGS cannot be judged or the patient’s infection status cannot be evaluated.

In detail, the judgment criteria for new-onset infections were based on the increase of body temperature after transplantation, continuously increased white blood cells, continuously increased procalcitonin (PCT), deteriorated sputum traits, hemodynamic instability, and CT scans by experienced lung transplant management experts.

### Statistical Analysis of Data

Descriptive statistics were computed for the overall sample and stratified by the presence of positive pathogen detected by NGS or bacterial culture positive on BALF samples. All statistical analyses were performed using the EXCEL software. Mean ± standard deviation (SD) or median (interquartile range, IQR) was used for describing the continuous variables.

## Results

### Clinical Characteristics of Patients in This Study

From October 2018 to July 2021, we completed 32 lung transplants in total, 30 cases of which were included in this study, including 26 men (86.67%) and 4 women (13.33%). The average age was 57.8 ± 1.03 years; the youngest recipient and the oldest recipient were 33 and 70 years old, respectively. Of the 30 lung transplantations, 15 were single-lung transplantations and 15 were bilateral lung transplantations. In this study, the primary disease of lung transplantation patients were interstitial lung disease (ILD) (50.0%), chronic obstructive pulmonary disease (COPD) (46.67%), and silicosis (3.33%). Other demographic details are listed in Table 1 and clinical characteristics of individuals in Table 2.

**TABLE 1:** Demographic and clinical characteristics of the 30 patients with lung transplantation.

Patient characteristics	Patients included (*n* = 30)
Male, n (%)	26 (86.67)
Age (years), mean (SD)	57.8 (1.03)
**Underlying condition, n (%)**	
ILD	15 (50.0)
COPD	14 (46.67)
Silicosis	1 (3.33)
**Type of transplant, n (%)**	
Single lung transplantation	15 (50.0)
Bilateral lung transplantation	15 (50.0)
**Other parameters (days)**	
MV time, median (interquartile range, IQR)	3 (IQR 13-1) (except 1 death case)
Length of stay in ICU, median (interquartile range, IQR)	8 (IQR 13-5) (except 6 death cases)

*ILD, interstitial lung disease; COPD, chronic obstructive pulmonary disease; MV, mechanical ventilation; ICU, intensive care unit.*

**TABLE 2:** Characteristics of individual transplant recipients.

	Age (years)	Gender	Type of transplant	ECLS	Initial antibiotics	Antibiotic regimen adjusted	Antibiotics after initial protocol changed	Antibiotic regimen adjustment	New-onset infections	MV time (days)	Days of stay in ICU (days)	Effect of the antibiotic adjustment strategy
Patient 1	70	Male	Single	No ECLS	Cefoperazone and sulbactam	Yes	Meropenem	II-3	Yes	2	9	None
Patient 2	69	Male	Bilateral	No ECLS	Piperacillin tazobactam	Yes	Piperacillin tazobactam, caspofungin	II-2	No	2	13	Positives
Patient 3	67	Male	Single	ECMO	Piperacillin tazobactam	Yes	Piperacillin tazobactam, vancomycin	II-3	Yes	15	NA	Positives
Patient 4	57	Male	Single	No ECLS	Cefoperazone and sulbactam	Yes	Meropenem	II-3	Yes	2	7	Positives
Patient 5	49	Male	Bilateral	No ECLS	Cefoperazone and sulbactam	No	Cefoperazone and sulbactam	I	No	1	8	Positives
Patient 6	52	Male	Single	No ECLS	Cefoperazone and sulbactam	Yes	Meropenem	II-2	No	1	10	Positives
Patient 7	50	Female	Single	No ECLS	Cefoperazone and sulbactam	Yes	Cefoperazone and sulbactam, compound sulfamethoxazole	II-2	No	1	13	Positives
Patient 8	67	Male	Single	No ECLS	Cefoperazone and sulbactam	No	Cefoperazone and sulbactam	I	No	3	6	Positives
Patient 9	61	Male	Bilateral	VV-ECMO	Cefoperazone and sulbactam	Yes	Piperacillin tazobactam, caspofungin	II-2	No	10	NA	Positives
Patient 10	54	Male	Single	VV-ECMO	Piperacillin tazobactam, Ganciclovir	No	Piperacillin tazobactam, ganciclovir	I	No	1	11	Positives
Patient 11	33	Male	Bilateral	No ECLS	Piperacillin tazobactam	Yes	Piperacillin tazobactam, voriconazole	II-2	No	1	5	Positives
Patient 12	46	Female	Single	VV-ECMO	Cefoperazone and sulbactam	No	Cefoperazone and sulbactam	I	No	1	7	Positives
Patient 13	64	Male	Single	VV-ECMO	Imipenem, vancomycin, Ganciclovir	No	Imipenem, vancomycin, ganciclovir	I	No	3	NA	Positives
Patient 14	60	Male	Bilateral	VV-ECMO	Cefoperazone and sulbactam, vancomycin	No	Cefoperazone and sulbactam, vancomycin	I	No	12	16	Positives
Patient 15	66	Female	Bilateral	VV-ECMO	Piperacillin tazobactam, vancomycin, Caspofungin	No	Piperacillin tazobactam, vancomycin, Caspofungin	I	No	25	NA	Positives
Patient 16	66	Male	Single	VV-ECMO	Cefoperazone and sulbactam	Yes	Meropenem, daptomycin, caspofungin	II-3	Yes	28	41	None
Patient 17	53	Male	Bilateral	VV-ECMO	Piperacillin tazobactam, ganciclovir	No	Piperacillin tazobactam, ganciclovir	I	No	1	3	Positives
Patient 18	53	Male	Bilateral	VV-ECMO	Cefoperazone and sulbactam	Yes	Cefoperazone and sulbactam, caspofungin	II-2	No	3	5	Positives
Patient 19	49	Male	Bilateral	VV-ECMO	Imipenem, linezolid, caspofungin acetate for injection	No	Imipenem, linezolid, caspofungin acetate	I	No	3	5	Positives
Patient 20	65	Male	Bilateral	VV-ECMO	Clindamycin, aztreonam	No	Clindamycin, aztreonam	I	No	49	NA	Positives
Patient 21	59	Male	Bilateral	VV-ECMO	Piperacillin tazobactam, linezolid	Yes	Piperacillin tazobactam	II-1	No	3	8	Positives
Patient 22	67	Male	Single	VV-ECMO	Piperacillin tazobactam, caspofungin	Yes	Imipenem, Linezolid, caspofungin	II-2	No	6	9	Positives
Patient 23	66	Male	Single	VV-ECMO	Cefoperazone and sulbactam	Yes	Cefoperazone and sulbactam, caspofungin	II-2	No	1	4	Positives
Patient 24	58	Male	Bilateral	VV-ECMO	Cefoperazone and sulbactam	Yes	Cefoperazone and sulbactam, moxifloxacin	II-3	Yes	4	8	Positives
Patient 25	64	Female	Single	VV-ECMO	Imipenem, vancomycin	No	Imipenem, vancomycin	I	No	19	21	Positives
Patient 26	61	Male	Bilateral	VV-ECMO	Piperacillin tazobactam, linezolid, isoniazid, ethambutol	Yes	Imipenem, Linezolid, isoniazid, Ethambutol	II-3	Yes	2	5	Positives
Patient 27	57	Male	Bilateral	VV-ECMO	Tegacyclin, caspofungin, linezolid, cefoperazone and sulbactam	Yes	Tegacyclin, caspofungin, linezolid, Sulbactam	II-3	Yes	NA	NA	Positives
Patient 28	62	Male	Bilateral	VV-ECMO	Linezolid, meropenem	Yes	Linezolid, meropenem, caspofungin	II-3	Yes	14	14	Positives
Patient 29	53	Male	Single	VV-ECMO	Moxifloxacin, caspofungin, piperacillin tazobactam	No	Moxifloxacin, caspofungin, piperacillin tazobactam	I	No	2	5	Positives
Patient 30	37	Male	Single	No ECLS	Moxifloxacin, caspofungin, piperacillin tazobactam	Yes	Imipenem, polymyxin, sulbactam, voriconazole	II-3	Yes	28	28	Negative

*ECLS, extracorporeal life support; VV ECMO, venous extracorporeal membrane oxygenation; ICU, intensive care unit; MV, mechanical ventilation; NA, not applicable.*

### Bronchoalveolar Lavage Fluid Samples Have the Highest Positive Rate Compared With Donor and Recipient Tissues

**FIGURE 1:**
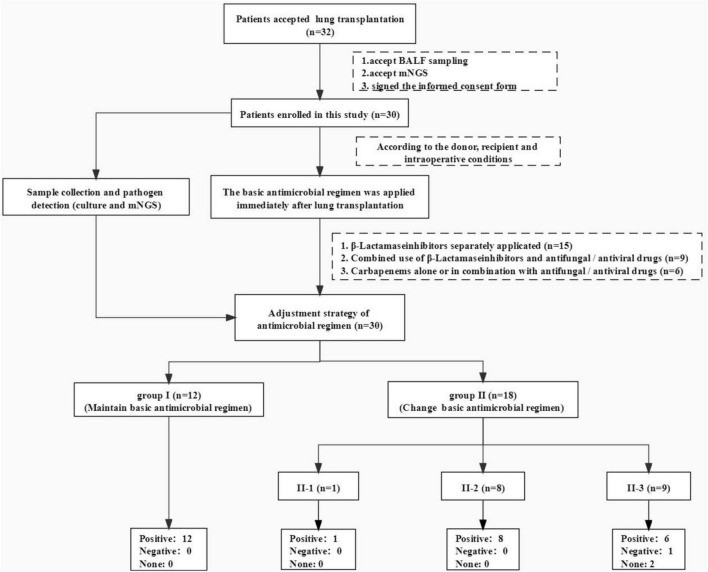
Flowchart of this study. We completed 32 lung transplants in total, 30 cases of which were included in this study, whose multiple clinical samples were sent to mNGS test and traditional culture at the same time. We prescribed those patients some basic antibiotic regimens (relatively narrow-spectrum antibiotics) immediately then made adjustments according to the clinical condition, pathogen test results (mNGS and pathogen culture of different samples), and experts’ opinions. At least two transplant management experts evaluated the effectiveness of our antibiotic adjustment strategy.

**FIGURE 2:**
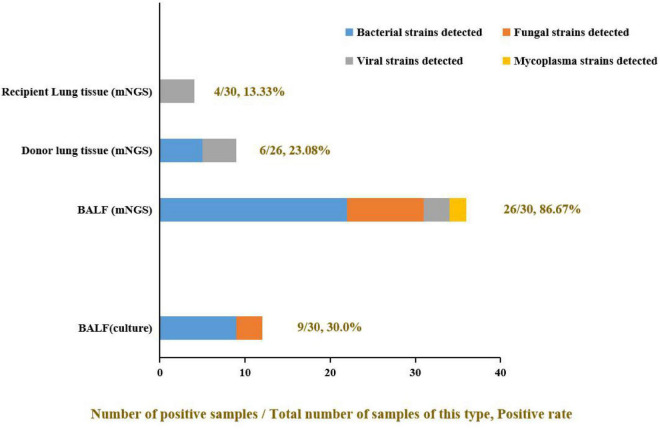
The positive rate of different samples and the proportion of pathogens detected. The positive rate of mNGS detection was significantly higher than in the pathogen culture of the same BALF samples (mNGS vs. culture: 86.67% vs. 30.0%). Some viruses and mycoplasma were detected by mNGS but not by culture method. Among all three kinds of clinical samples, BALF samples showed the highest clinical pathogen diagnostic value: only virus detected in recipient lung tissue; only virus and mycoplasma detected in donor lung tissue, but most kinds of pathogens were detected in BALF and the bacteria made up the majority.

The donor lung tissue, recipient lung tissue, and BALF samples were submitted for pathogen culture and mNGS according to the study flow chart (Figure 1) after lung transplantation. A total of 86 effective samples were obtained in this study; the BALF test showed the highest positive rate (86.67%, 26/30) by mNGS. Meanwhile, the positive rate of pathogen culture on the BALF was only 36.67% (11/30). In detail, pathogen cultures on nine BALF samples (30%, 9/30) grew out bacteria such as *Staphylococcus aureus*, *Staphylococcus haemolyticus*, *Burkholderia multivorans*, *Acinetobacter ursingii*, and *Klebsiella pneumoniae*; only one BALF sample (0.33%, 1/30) grew fungal (*Candida parapsilosis*). But there were 22 bacteria, 9 fungi, 3 viruses, and 2 mycoplasma detected by mNGS for the same BALF samples, and for the 26 donor and 30 recipient lung tissue samples, there were fewer pathogens tested in their mNGS reports (Figure 2), which may suggest that such samples are not well correlated with infection after lung transplantation. Thus, BALF may be the most appropriate sample for pathogen detection after lung transplantation.

### The Pathogenic Spectrum of Bronchoalveolar Lavage Fluid Detected by mNGS in This Study

**FIGURE 3:**
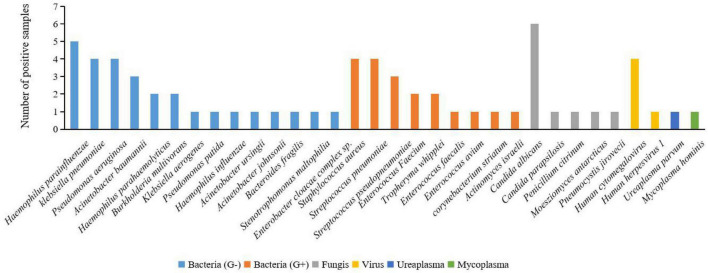
Positive pathogen spectrum detected in BALF samples by mNGS in this study. Of all the bacteria detected by mNGS in BALF samples, G-bacteria (*Haemophilus parainfluenzae*, *klebsiella pneumoniae*, and *Pseudomonas aeruginosa*) made up the majority. Several rare pathogens were also detected in the BALF samples, such as *Penicillium citrinum*, *Ureaplasma parvum*, and *Mycoplasma hominis*.

To clear all possible pathogens after lung transplantation, we used mNGS to make the pathogen spectrum of BALF. According to the mNGS results of BALF (Figure 3), 22 bacteria were detected in 30 samples, including Gram-negative bacteria (66.67%, 20/30), such as *Haemophilus*, *klebsiella*, *Pseudomonas*, *Acinetobacter*, *Burkholderia*, *Bacteroides*, *Stenotrophomonas*, and *Enterobacter*. Gram-positive bacteria (36.67%, 11/30) included *Staphylococcus, Enterococcus, Tropheryma, corynebacterium*, and *Actinomyces*. Also, there were fungi (16.67%, 5/30) like *Candida*, *Penicillium*, *Moesziomyces*, and *Pneumocystis*. There were two pathogenic viruses detected in four BALF samples (13.33%, 4/30): *Human cytomegalovirus* and *Human herpesvirus 1*. Some rare pathogens were also discovered such as *Ureaplasma parvum* (0.33%, 1/30) and *Mycoplasma hominis* (0.33%, 1/30). This indicated that Gram-negative bacteria may be the first pathogen to be considered after lung transplantation.

### Efficacy of Our Antibiotic Adjustment Strategies in This Study

Of the patients in this study, 50% (15/30) used β-lactamase inhibitor as the only antibiotic; 30.0% (9/30) of patients were administered with combined β-lactamase inhibitor and some other anti-fungal or anti-viral drugs, such as Caspofungin, voriconazole, cotrimoxazole, and ganciclovir, or other anti-microbial drugs such as moxifloxacin and vancomycin. The other 20.0% (6/30) of patients use carbapenems (amopenem or meropenem) that replaced β-lactamase inhibitors.

The basic antibiotic regimen was adjusted according to the pathogen detection reports from pathogen culture and mNGS and also the patient’s clinical indications. After the strategy application, 40.0% (12/30) of patients maintained the initial medication regimen because it could cover the pathogen in the comprehensive clinical situation within 7 days after transplantation. The other 60.0% (18/30) of patients adjusted the antibiotic regimen, in which:

We reduced the antibiotic in one patient, whose basic antibiotic plan was piperacillin/tazobactam combined with linezolid. *Pseudomonas aeruginosa* and *Pseudomonas putida* were the main pathogens according to mNGS, so the linezolid was suspended. This patient had no new-onset infection within 7 days after prognosis, and the effect of the strategy was positive.

Eight patients had no new-onset infections within 7 days, but their antibiotic regimen was changed because lung transplant management experts predicted that there will occur infections according to the clinical situation and mNGS reports. Also, the effect of our strategy in the eight cases was all positive.

The remaining 9 patients had progressed infection status, so antibiotics regimens were adjusted for suspected pathogens mainly depending on reports of mNGS. In this study, most cases were supplemented with corresponding antibiotics for a possible fungal infection or viral infection. According to the effect evaluation results, the effect of the strategy in most cases (66.67%, 6/9) was positive; just one case (11.11%) was negative in which infection status cannot be controlled. The effect of the strategy in the last two patients cannot be evaluated because antibiotic adjustment did not refer to their mNGS reports.

According to the comprehensive judgment of professional clinical management experts of lung transplantation, using the antibiotic adjustment strategies after lung transplantation combined with mNGS and pathogen culture, 70.0% (21/30) of patients in this study did not get new-onset infections within 7 days after transplantation; in 6.67% (2/30) of patients, infection reduced; in 13.33% (4/30), infection was effectively controlled; in 3.33% (1/30), infection was aggravated and the strategies failed; and in 6.67% (2/30), the antibiotic regimen was adjusted but not referring to the mNGS reports. In conclusion, the entire effectiveness of this strategy was 90.0% (27/30). Therefore, our antibiotic adjustment strategy has the prospect of further application in the management of clinical lung transplantation.

## Discussion

In this study, we made a new antibiotic regimen adjustment strategy in the very early stage (within 7 days) after lung transplantation mainly depending on pathogenic test results of different clinical samples and patients’ clinical indications. We confirmed that the effectiveness of this strategy was 90.0% through 30 lung transplantation cases.

Half of the infectious episodes following lung transplantation occur in the first 30 days (Aguilar-Guisado et al., 2007), but it is not clear which period occurred infectious within 30 days. The data related to the infection in the very early stage (within 7 days) after lung transplantation is limited (Parada et al., 2010). In our study, of the 30 patients enrolled in this study, 10 (33.33%, 10/30) patients developed a pulmonary infection within 1 week after the operation. Among them, 7 patients (70%, 7/10) developed the infection within 72 h after the operation, indicating that early postoperative pulmonary infection (within 7 days) cannot be ignored.

A previous study (Liu et al., 2020) suggested that the colonized bacteria in different parts of the lung are inconsistent and there is no association between the colonized bacteria in donor lungs and the short-term outcome of lung transplantation patients. In our study, the positive rate by mNGS test of BALF samples was higher (86.67%) than that of donor lung tissue (20.0%) or recipient lung tissue (13.33%). It indicates BALF samples from the early postoperative period are more valuable than other clinical samples to guide the early adjustment of antibiotics after lung transplantation. Meanwhile, it is the first study about the value of pathogen detection of different clinical samples in the adjustment of the anti-infection scheme after lung transplantation.

The pathogens of pulmonary infection in the early stage after transplantation are complex (Nosotti et al., 2018; José et al., 2020). Our study suggested that the proportion of Gram-negative bacteria and Gram-positive bacteria detected by mNGS in the BALF of lung transplant recipients was 66.67 and 33.33%, respectively. The significance is that Gram-negative bacteria are still the pathogen that needs to be covered first when the pathogen is unknown. The positive rate of fungi (33.33%) also suggested that the prevention of fungal infection after lung transplantation is worthy of attention.

The postoperative antimicrobial strategies adopted by many centers are often for the sake of wide coverage of all possible pathogens (Coiffard et al., 2020). For example, the scheme (Ling et al., 2016) of “meropenem, vancomycin, caspofungin, and ganciclovir were used simultaneously” was adopted by Guangzhou Institute of Respiratory Diseases in China. In our study, we developed a new strategy for super early-stage (within 7 days) antibiotic optimization after lung transplantation: donor lung tissue, recipient lung tissue, and BALF within 2 h after surgery were all sent to mNGS (BALF also sent to pathogen culture); we gave the patients a basic antibiotic regimen (relatively narrow spectrum) and optimized the regimen according to their clinical indications and pathogen detection results after a relevant pathogen detection report was obtained, of which the effective rate is 90% (27/30). It suggested that not only is our antibiotic optimization strategy effective, but also, it is not necessary to use so many antibiotics which may increase the chance of multi-drug-resistant bacteria, the economic burden of patients, and also the liver and kidney function injury of patients.

The average mechanical ventilation time of the patients enrolled in this study was 3 (IQR 13-1) days (excluding 1 death), the average ICU hospitalization time was 8 (IQR 13-5) days (excluding 6 deaths), and the 30-day mortality was 80.0% (24/30). Liu’s study on the application of mNGS in the management of lung transplantation in Wuxi People’s Hospital showed that 17 patients were included in the study, and the average mechanical ventilation time was 73.3 (SD = 67.6) h, the average ICU hospitalization time was 112.6 (SD = 59.8) h, and the average hospital stay was 41.2 (SD = 46.9) days. Our strategy reduced the unnecessary postoperative antibiotics of wide coverage while it did not increase the incidence of infection, MV time, and ICU length of stay.

In this study, the strategy of adjusting antibiotics after lung transplantation based on traditional pathogen detection combined with mNGS is effective for the prevention and control of new-onset infections. However, due to the high difficulty of lung transplantation and less clinical practice in China, the number of cases in this study is small (30 cases totally). Therefore, there is no distinction between the types of primary diseases (such as pulmonary interstitial disease or COPD) and lung transplantation methods (such as single lung or bilateral lung transplantation). In addition, because of the particularity of lung transplantation, a specific control group cannot be set in this study, but the effect of our strategy can be judged by comparing it with other studies. Finally, how this antibiotic optimization strategy works in different clinical situations and how it affects the long-term prognosis of lung transplantation patients should be verified by the clinical practice of larger population applications.

## Conclusion

It is effective to apply mNGS combined with traditional pathogen detecting methods and clinical features to optimize antibiotic regimens in lung transplantation recipients within 7 days after surgery. For patients who accepted lung transplantation, BALF samples from the early postoperative period are more valuable than other clinical samples to guide the early adjustment of antibiotics.

## Data Availability Statement

The raw data supporting the conclusions of this article will be made available by the authors, without undue reservation.

## Ethics Statement

The studies involving human participants were reviewed and approved by Sichuan Provincial People’s Hospital. The patients/participants provided their written informed consent to participate in this study.

## Author Contributions

XZ and SL designed and performed the study. XZ, XT, YL, HY, and LP collected and analyzed data. XT, XZ, and LP wrote the manuscript. SL, LP, and YW supervised the clinical research and revised the manuscript. All authors approved the final manuscript.

## Conflict of Interest

XT is employed by Genoxor Medical Science and Technology Inc., Taizhou, China. The remaining authors declare that the research was conducted in the absence of any commercial or financial relationships that could be construed as a potential conflict of interest.

## Publisher’s Note

All claims expressed in this article are solely those of the authors and do not necessarily represent those of their affiliated organizations, or those of the publisher, the editors and the reviewers. Any product that may be evaluated in this article, or claim that may be made by its manufacturer, is not guaranteed or endorsed by the publisher.
